# Supplementary figures and images for: Tracking changes between preprint posting and journal publication during a pandemic
Source: PLoS Biol. 2022 Feb 1;20(2):e3001285. doi: 10.1371/journal.pbio.3001285 (PMC8806067; doi:10.1371/journal.pbio.3001285)

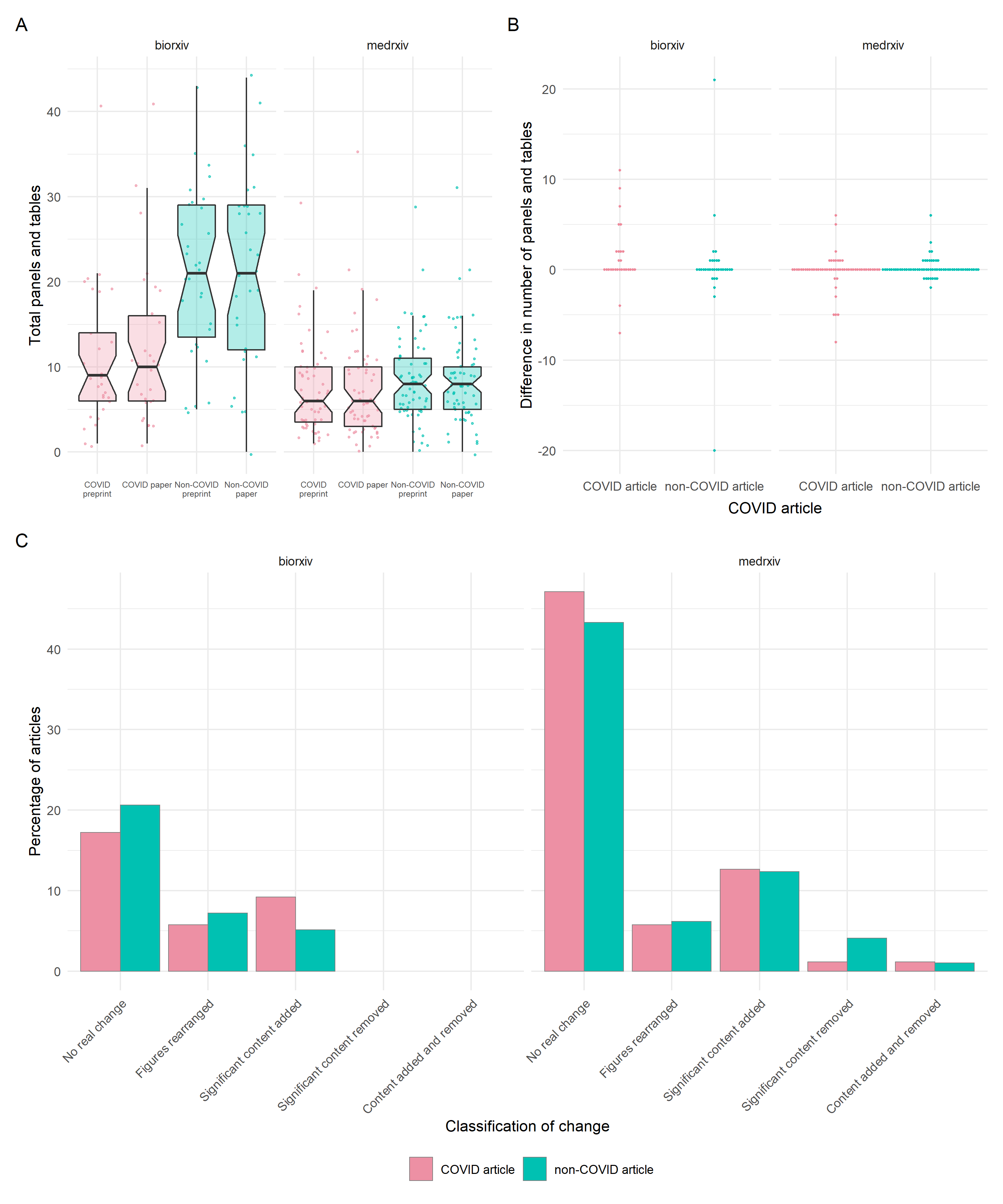

Supplement: S2 Fig — (A) Total numbers of panels and tables in preprints and published articles. Boxplot notches denote approximated 95% CI around medians. (B) Difference in the total number of panels and tables between the preprint and published versions of articles. (C) Classification of figure changes between preprint and published articles. The data underlying this figure may be found at https://github.com/preprinting-a-pandemic/preprint_changes and https://zenodo.org/record/5594903#.YXUv9_nTUuU. CI, confidence interval. (TIFF) [file pbio.3001285.s002.tiff]

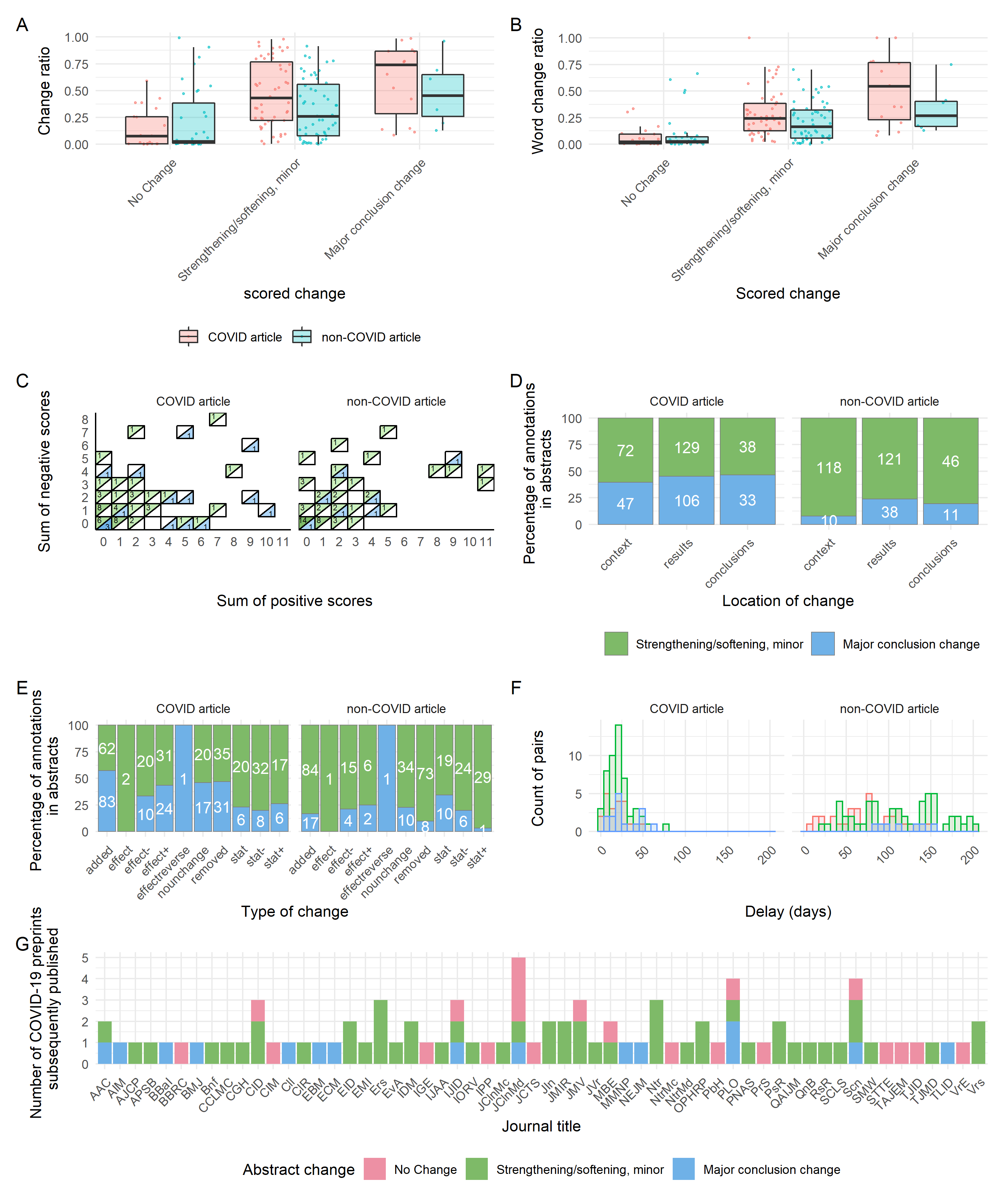

Supplement: S3 Fig — (A) Difflib calculated change ratio for COVID-19 or non-COVID-19 abstracts, based on the overall abstract change. (B) Change ratio calculated from Microsoft Word for COVID-19 or non-COVID-19 abstracts, based on the overall abstract change. (C) Sum of positive and negative annotations based on the overall abstract change, with colour and label denoting number of abstracts with each particular sum combination. A total of 21 COVID-19 preprints and 35 non-COVID-19 preprints rated “No change” (i.e., sum of positive and negative scores = 0) are not depicted. (D) Percentage of annotations in each location within COVID-19 or non-COVID-19 abstracts, based on the overall abstract change. Labels denote absolute number of annotations. (E) Percentage of annotations of each type within COVID-19 or non-COVID-19 abstracts, based on the overall abstract change. Labels denote absolute number of annotations. (F) Delay (in days) between preprint posting and publication in a journal, based on overall abstract changes. (G) Journals publishing COVID-19 preprints, based on overall abstract changes. See S1 Text for key to abbreviated journal labels. The data underlying this figure may be found at https://github.com/preprinting-a-pandemic/preprint_changes and https://zenodo.org/record/5594903#.YXUv9_nTUuU. COVID-19, Coronavirus Disease 2019. (TIFF) [file pbio.3001285.s003.tiff]

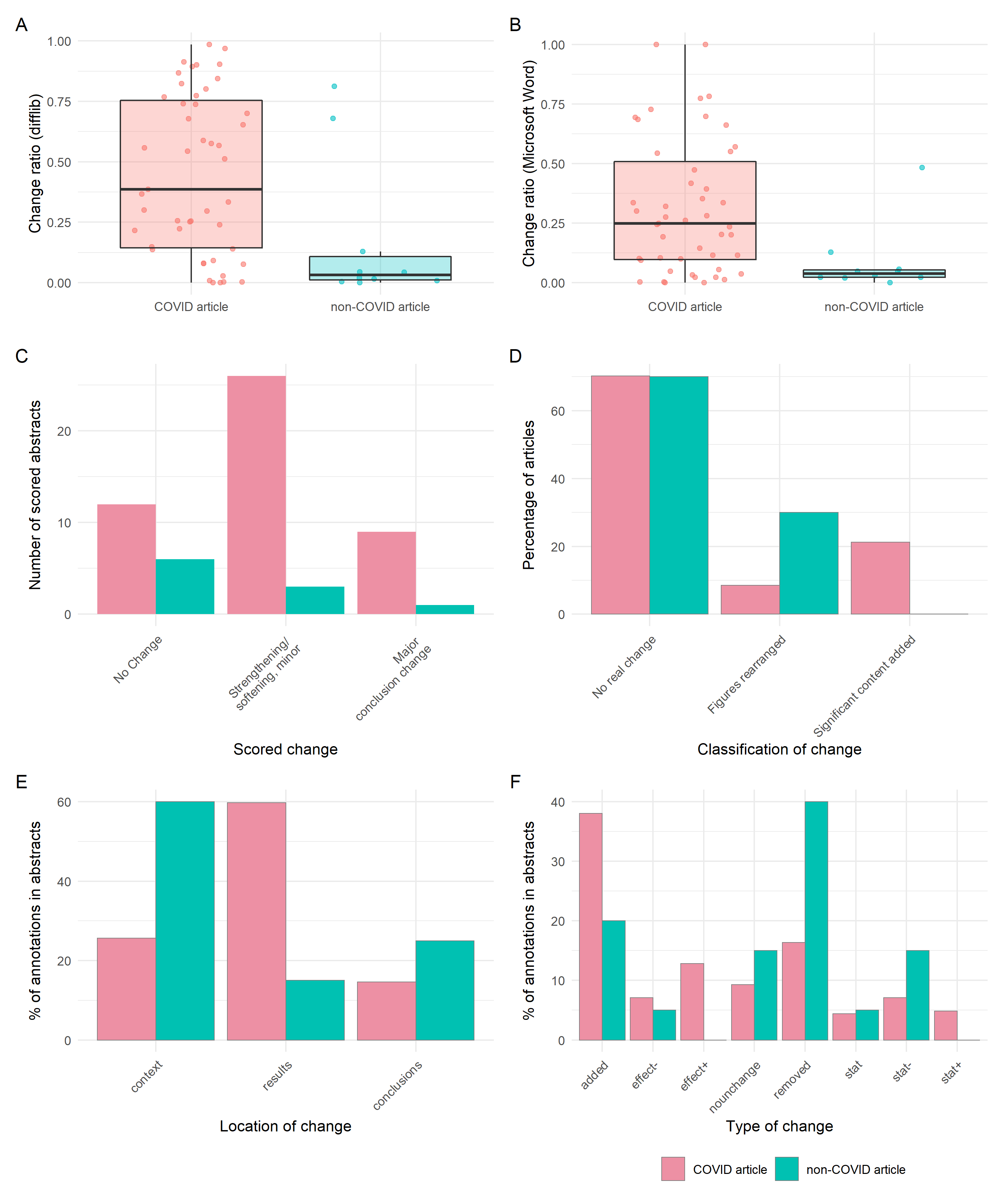

Supplement: S4 Fig — (A) Difflib calculated change ratio for COVID-19 or non-COVID-19 abstracts. (B) Change ratio calculated from Microsoft Word for COVID-19 or non-COVID-19 abstracts. (C) Overall changes in abstracts for COVID-19 or non-COVID-19 abstracts. (D) Classification of figure changes between preprint and published articles for COVID-19 or non-COVID-19 abstracts. (E) Location of annotations within COVID-19 or non-COVID-19 abstracts. (F) Type of annotated change within COVID-19 or non-COVID-19 abstracts. The data underlying this figure may be found at https://github.com/preprinting-a-pandemic/preprint_changes and https://zenodo.org/record/5594903#.YXUv9_nTUuU. COVID-19, Coronavirus Disease 2019. (TIFF) [file pbio.3001285.s004.tiff]
