# Supplementary material for: Tracking changes between preprint posting and journal publication during a pandemic
Source: PLoS Biol. 2022 Feb 1;20(2):e3001285. doi: 10.1371/journal.pbio.3001285 (PMC8806067; doi:10.1371/journal.pbio.3001285)
Supplement: S2 Table — (DOCX) [file pbio.3001285.s006.docx]

### Supplementary table 2. Examples of changes in abstracts between the preprint and published version of an article

###

| **Example** | **Preprint DOI** | **Change (strikethrough: removed, bold: added)**  *Note that artifacts introduced by Word, such as duplicate numbers and symbols, have been removed.* | **Full annotation** |
| --- | --- | --- | --- |
| Added | 10.1101/2020.02.29.20029322 | To address this concern, the study employed a total of 214 general public ~~(GP)~~ and 526 nurses **(i.e., 234 front-line nurses and 292 non-front-line nurses)** to evaluate ~~VT~~ **vicarious traumatization** scores via a mobile app-based questionnaire. | Context_added_1 |
| Removed | 10.1101/19006478 | Multifocal **(MF)/multicentric (MC))** breast cancer is generally considered to be where two or more breast tumours are present within the same breast, ~~but are clearly separated with no intervening in situ or invasive disease.~~ | Context_removed_1 |
| Noun change | 10.1101/19009589 | We found that transportation potential was higher for source countries with seasonal dengue activity, high passenger traffic, high incidence rates, ~~lower economic status~~ **high epidemic vulnerability**, and in geographical proximity to a destination country in Europe. | Results_nounchange_1 |
| Effect + | 10.1101/2020.03.09.983247 | EK1C4 was also highly effective against membrane fusion and infection of other human coronavirus pseudoviruses tested, including SARS-CoV and MERS-CoV, as well as SARSr-CoVs, and potently ~~inhibiting~~ **inhibited the** replication of ~~4~~ **5** live human coronaviruses examined, including SARS-CoV-2. | Results_effect+_1 |
| Effect - | 10.1101/2020.01.23.916395 | The mean estimate of R0 for the 2019-nCoV ranges from ~~3.30 (95%CI: 2.73-3.96) to 5.47 (95%CI: 4.16-7.10)~~,**2.24 to 3.58** , and **is** significantly larger than 1 | Results_effect-_1 |
| Stat+ | 10.1101/19010975 | Logistic regression AUCs were 0.~~82~~**83**, 0.~~82~~**83**, 0.~~79~~**81** for three different regularization schemes; tree boosting AUC was 0.81; MLP AUC was 0.~~81~~**83** | Results_stat+_1+ |
| Stat+ (wording) | 10.1101/19006031 | ~~These findings reveal a modest negative association .:~~ **Findings describe small but robust associations** between lifetime MDD and **lower** cognitive performance. within a population-based sample. | Conclusions_stat+_1+ |
| Stat- | 10.1101/19011064 | Greater paternal engagement (OR 1.~~59~~**5** (1.~~13~~**09**, 2.~~21~~**07**) was associated with [...] | Results_stat-_1- |
| Stat- (wording) | 10.1101/19005710 | Elevated STE from 5- to 19 year-olds indicates that school-aged children were **likely** the most important transmitters of infection during the autumn wave of the 2009 pandemic in the US**A.** | Conclusion_stat-_1- |
| Statinfo | 10.1101/19007013 | The ~~MR~~ **Mendelian randomisation** analysis and **single** ~~SNP~~-**nucleotide polymorphism** analysis, however, did not support this**. (odds ratio for lifetime smoking on suicidal ideation, 0.050; 95% CI -0.027 to 0.127; odds ratio on suicide attempts, 0.053; 95% CI, -0.003 to 0.110)**. | Results_statinfo_1+ |

### 
