# Supplementary material for: Tracking changes between preprint posting and journal publication during a pandemic
Source: PLoS Biol. 2022 Feb 1;20(2):e3001285. doi: 10.1371/journal.pbio.3001285 (PMC8806067; doi:10.1371/journal.pbio.3001285)
Supplement: S1 Method — (PDF) [file pbio.3001285.s010.pdf]

Preprint-->paper evaluation form

* Required

1. Who are you? *

Mark only one oval.

- ⬭ JC
- ⬭ MP
- ⬭ GD
- ⬭ JP

2. Which dataset? *

Mark only one oval.

- ⬭ COVID
- ⬭ Control

3. Manuscript number (according to sheet) *

____

4. Re-enter manuscript number (according to sheet) *

____

5. Has the author list changed?

Check all that apply.

- [ ] No
- [ ] Yes, authors added
- [ ] Yes, authors removed
- [ ] Yes, change in corresponding/co-corresponding authors

6. Has the abstract changed?

Mark only one oval.

- ( ) No change or minor: change in wording, if any, does not change main conclusion(s)
- ( ) Yes, significant: altered wording or numbers leading to a softening/strengthening of main conclusions(s)
- ( ) Yes, major: a discrete change in the main conclusion(s)
- ( ) Yes, massive: main conclusion(s) of the paper reversed

7. How many figures/panels in the preprint (not supplement)?

Check all that apply.

	1 panel	2 panels	3 panels	4 panels	5 panels	6 panels	7 panels	8 panels	9 pane
Figure 1	☐	☐	☐	☐	☐	☐	☐	☐	☐
Figure 2	☐	☐	☐	☐	☐	☐	☐	☐	☐
Figure 3	☐	☐	☐	☐	☐	☐	☐	☐	☐
Figure 4	☐	☐	☐	☐	☐	☐	☐	☐	☐
Figure 5	☐	☐	☐	☐	☐	☐	☐	☐	☐
Figure 6	☐	☐	☐	☐	☐	☐	☐	☐	☐
Figure 7	☐	☐	☐	☐	☐	☐	☐	☐	☐
Figure 8	☐	☐	☐	☐	☐	☐	☐	☐	☐
Figure 9	☐	☐	☐	☐	☐	☐	☐	☐	☐
Figure 10 or more (combine additional)	☐	☐	☐	☐	☐	☐	☐	☐	☐

8. How many tables in the preprint (not supplement)?

Mark only one oval.

	0	1	2	3	4	5	
	☐	☐	☐	☐	☐	☐	Choose 5 for >=5

9. How many figures/panels in the main paper (not supplement)?

Check all that apply.

	1 panel	2 panels	3 panels	4 panels	5 panels	6 panels	7 panels	8 panels	9 pane
Figure 1	☐	☐	☐	☐	☐	☐	☐	☐	☐
Figure 2	☐	☐	☐	☐	☐	☐	☐	☐	☐
Figure 3	☐	☐	☐	☐	☐	☐	☐	☐	☐
Figure 4	☐	☐	☐	☐	☐	☐	☐	☐	☐
Figure 5	☐	☐	☐	☐	☐	☐	☐	☐	☐
Figure 6	☐	☐	☐	☐	☐	☐	☐	☐	☐
Figure 7	☐	☐	☐	☐	☐	☐	☐	☐	☐
Figure 8	☐	☐	☐	☐	☐	☐	☐	☐	☐
Figure 9	☐	☐	☐	☐	☐	☐	☐	☐	☐
Figure 10 or more (combine additional)	☐	☐	☐	☐	☐	☐	☐	☐	☐

10. How many tables in the paper (not supplement)?

Mark only one oval.

	0	1	2	3	4	5	
	☐	☐	☐	☐	☐	☐	Choose 5 for >=5

11. Does the change between preprint and paper in the main figures (including tables) reflect a change in content or outcomes? *

Check all that apply.

- [ ] No, no real changes at all (including reorganisation)
- [ ] No, panels or tables have just been moved around (including to supplement if available)
- [ ] Yes, significant additional content/outcomes have been added
- [ ] Yes, significant content/outcomes have been removed

12. Does the preprint have a supplement? How many files or items (figures, spreadsheets, extended methods)?

Mark only one oval.

	0	1	2	3	4	5	
	○	○	○	○	○	○	Select option 5 for >=5

13. If the preprint does have a supplement, does it contain figures?

Mark only one oval.

- ○ Yes
- ○ No

14. Does the paper have a supplement? How many files or items (figures, spreadsheets, extended methods)?

Mark only one oval.

	0	1	2	3	4	5	
	○	○	○	○	○	○	Select option 5 for >=5

15. If the paper does have a supplement, does it contain figures?

Mark only one oval.

- ( ) Yes
- ( ) No

16. Is the source data (including code) more accessible after publication?

Check all that apply.

- [ ] No, same as preprint - available only upon request
- [ ] No, same as the preprint - available through repositories or supplementary files
- [ ] Yes, provided as additional supplementary files
- [ ] Yes, provided through repositories

Other: [ ] ____________________

17. Are open peer reviews and decision letters available?

Mark only one oval.

- ( ) Yes, via Review Commons or any other post-publication review scheme
- ( ) Yes, provided by the journal
- ( ) No
- ( ) Don't know/ cannot locate this information

18. Any other comments?

____

____

____

____

____
