# Supplementary material for: Tracking changes between preprint posting and journal publication during a pandemic
Source: PLoS Biol. 2022 Feb 1;20(2):e3001285. doi: 10.1371/journal.pbio.3001285 (PMC8806067; doi:10.1371/journal.pbio.3001285)
Supplement: S1 Text — (DOCX) [file pbio.3001285.s013.docx]

| **Journal title** | **Abbreviated label** |
| --- | --- |
| 3 Biotech | 3Bt |
| ACS Applied Bio Materials | AABM |
| Antimicrobial Agents and Chemotherapy | AAC |
| Acta Biomaterialia | AcB |
| Archives of Iranian Medicine | AIM |
| American Journal of Clinical Pathology | AJCP |
| Acta Neuropathologica Communications | ANC |
| Archives of Public Health | APH |
| Acta Pharmaceutica Sinica B | APSB |
| Brain, Behavior, and Immunity | BBaI |
| Biochemical and Biophysical Research Communications | BBRC |
| Biochemistry | Bch |
| BMC Endocrine Disorders | BED |
| Biogerontology | Bgr |
| Blood | Bld |
| BMC Genomics | BMG |
| BMJ | BMJ |
| BMJ Open | BMO |
| BMC Pediatrics | BMP |
| Bioinformatics | Bnf |
| Brain | Brn |
| Clinical Chemistry and Laboratory Medicine (CCLM) | CCLMC |
| Clinical Gastroenterology and Hepatology | CGH |
| Clinical Infectious Diseases | CID |
| Clinical Immunology | CIM |
| Clinical Epigenetics | ClE |
| Cell | Cll |
| Cell Reports | CllRp |
| Cell Research | CllRs |
| Clinical Pharmacokinetics | ClP |
| Cancers | Cnc |
| ClinicoEconomics and Outcomes Research | COR |
| Developmental Biology | DvB |
| Diversity and Distributions | DvD |
| EBioMedicine | EBM |
| EClinicalMedicine | ECM |
| Emerging Infectious Diseases | EID |
| eLife | eLf |
| Emerging Microbes & Infections | EMI |
| Epidemics | Epd |
| European Psychiatry | ErP |
| Eurosurveillance | Ers |
| ESMO Open | ESO |
| Evolutionary Applications | EvA |
| EvoDevo | EvD |
| Frontiers in Integrative Neuroscience | FIN |
| Frontiers in Plant Science | FPS |
| Genetics in Medicine | GnM |
| Genetics | Gnt |
| Heart Rhythm | HrR |
| Infectious Disease Modelling | IDM |
| Infection, Genetics and Evolution | IGE |
| International Journal of Antimicrobial Agents | IJAA |
| IEEE Journal of Biomedical and Health Informatics | IJBHI |
| International Journal of Behavioral Nutrition and Physical Activity | IJBNPA |
| International Journal of Cancer | IJC |
| International Journal of Environmental Research and Public Health | IJERPH |
| International Journal for Parasitology | IJfP |
| International Journal of Infectious Diseases | IJID |
| Influenza and Other Respiratory Viruses | IORV |
| Infection Prevention in Practice | IPP |
| Journal of Allergy and Clinical Immunology | JAC |
| Journal of Clinical Microbiology | JClnMc |
| Journal of Clinical Medicine | JClnMd |
| Journal of Clinical Pathology | JCP |
| Journal of Clinical and Translational Science | JCTS |
| Journal of the Intensive Care Society | JICS |
| Journal of Infection | JIn |
| JMIR Medical Informatics | JMI |
| Journal of Medical Internet Research | JMIR |
| Journal of Medical Virology | JMV |
| Journal of Neurology, Neurosurgery & Psychiatry | JNNP |
| Journal of Neurology | JNr |
| Journal of Public Health | JPH |
| Journal of Psychopharmacology | JPs |
| Journal of The Royal Society Interface | JTRSI |
| Journal of Virology | JVr |
| Molecular Biology of the Cell | MBC |
| Mathematical Biosciences and Engineering | MBE |
| mBio | mBi |
| Molecular Biology Reports | MBR |
| Molecular & Cellular Proteomics | MCP |
| Molecular Autism | MlA |
| Mathematical Modelling of Natural Phenomena | MMNP |
| Multiple Sclerosis Journal | MSJ |
| mSphere | mSp |
| Metabolism | Mtb |
| Nucleic Acids Research | NAR |
| New England Journal of Medicine | NEJM |
| NeuroImage: Clinical | NIC |
| Nature Machine Intelligence | NMI |
| Neurology - Neuroimmunology Neuroinflammation | NNN |
| Neurobiology of Aging | NrA |
| Neurology | Nrl |
| Nature Communications | NtC |
| Nature | Ntr |
| Nature Microbiology | NtrMc |
| Nature Medicine | NtrMd |
| Open Forum Infectious Diseases | OFID |
| Osong Public Health and Research Perspectives | OPHRP |
| Public Health | PbH |
| Polar Biology | PlB |
| PLOS ONE | PLO |
| Proceedings of the National Academy of Sciences | PNAS |
| Progress in Neuro-Psychopharmacology and Biological Psychiatry | PNPBP |
| PLOS Neglected Tropical Diseases | PNTD |
| Protein Science | PrS |
| Psychological Medicine | PsM |
| Psychiatry Research | PsR |
| QJM: An International Journal of Medicine | QAIJM |
| Quantitative Biology | QnB |
| Respiratory Research | RsR |
| Supportive Care in Cancer | SCC |
| Science China Life Sciences | SCLS |
| Science | Scn |
| Swiss Medical Weekly | SMW |
| Science of The Total Environment | STTE |
| The American Journal of Emergency Medicine | TAJEM |
| Therapeutic Advances in Neurological Disorders | TAND |
| The British Journal of Psychiatry | TBJP |
| The EMBO Journal | TEJ |
| The Journal of Infectious Diseases | TJID |
| The Journal of Molecular Diagnostics | TJMD |
| The Journal of Pathology: Clinical Research | TJPCR |
| The Lancet Infectious Diseases | TLID |
| Translational Psychiatry | TrP |
| Vaccine | Vcc |
| Virus Evolution | VrE |
| Viruses | Vrs |
