# Supplementary material for: Tracking changes between preprint posting and journal publication during a pandemic
Source: PLoS Biol. 2022 Feb 1;20(2):e3001285. doi: 10.1371/journal.pbio.3001285 (PMC8806067; doi:10.1371/journal.pbio.3001285)
Supplement: S1 Appendix — (DOCX) [file pbio.3001285.s014.docx]

**Appendix S1. Illustrative power calculations for Chi-square and correlation tests.**

Our sample size of 184 preprint-paper pairs in this study were limited by the intensive manual inspection and annotation required. For illustration, we provide calculations of statistical power of Chi-square and correlation tests to detect a given effect size, given our sample size and assuming the effect is present, i.e., 1 – probability of a Type II error assuming the null hypothesis is false (hereafter “power”). Power is calculated for “small”, “medium”, and “large” benchmarked effect sizes as defined by Cohen [1]. Power for Chi-square tests is calculated for cases with df = 1 (i.e., a 2x2 contingency table). All power calculations were conducted with R package `pwr`, v1.3.0 [2]. With the given sample size, power to detect “medium” effect sizes was consistently > 0.98 (Appendix Table 1), though power to detect “small” effect sizes was much lower.

| Effect size (Cohen 1992) | Power  (Chi-square test, *w*) | Power  (Correlation test, *r*) |
| --- | --- | --- |
| small | 0.2735 | 0.2723 |
| medium | 0.9825 | 0.9866 |
| large | >0.9999 | >0.9999 |

**Appendix Table 1. Calculated statistical power for Chi-square and correlation tests given a sample size of n = 184 and significance level (alpha) = 0.05. Italics denotes the specific measure of effect size used for each respective test.**

**References**

1. Cohen J. A power primer. Psychol Bull. 1992;112: 155–159. doi:10.1037//0033-2909.112.1.155

2. Champely S. pwr: Basic Functions for Power Analysis. R package version 1.3-0. 2020. Available: https://CRAN.R-project.org/package=pwr
