# Supplementary material for: Tracking changes between preprint posting and journal publication during a pandemic
Source: PLoS Biol. 2022 Feb 1;20(2):e3001285. doi: 10.1371/journal.pbio.3001285 (PMC8806067; doi:10.1371/journal.pbio.3001285)
Supplement: Attachment — Submitted filename: Reviewer Comments.docx [file pbio.3001285.s015.docx]

Dear Editor,

Thank you for the opportunity to revise our manuscript. Please find below point-by-point responses to each of the comments made by the reviewers.

We have altered the title and our wording throughout the manuscript to better reflect our focus on the early phase of the pandemic. We have also performed extensive statistical analyses throughout to better support our data and conclusions.

Kind regards

*****************************************************

REVIEWERS' COMMENTS:


Reviewer #1:
[identifies himself as Ross Mounce]

This manuscript 'Preprints in motion: tracking changes between posting and journal publication' presents an analysis of (only bioRxiv and medRxiv) 14,812 preprints that had been posted between 1st January and 30th April 2020. They then identify which of these subsequently can be identified as published in a journal and classify them as either COVID-19 or not COVID-19 related. They then attempt to compare n=105 COVID-19 related preprint-paper pairs with n=105 not COVID-19 related preprint-paper pairs to discern changes between preprint and journal-published versions, and to see if there are any differences between the COVID-19 related and not COVID-19 related sets. They focus on textual changes in abstracts, article level metrics, and on the number of figures and tables between preprint and journal-published versions of a work. The results are newsworthy e.g. "the main conclusions [in abstracts] of 94% of non-COVID-related life sciences articles do not change from their preprint to final published versions".

I think it's an interesting manuscript worthy of publication in PLOS Biology after a few relatively minor revisions.
I thank for the authors for putting the underlying code and data on github (https://github.com/preprinting-a-pandemic/preprint_changes) AND for preserving a proper archive snapshot of that material on Zenodo. Great practice.

We would like to thank the reviewer for their time and thoughtful comments.

**Comments on the abstract of this manuscript**

In my opinion the abstract of this manuscript contains a debatable assertion which I strongly disagree with: the last line states "…but the majority of these changes do not reverse the main message of the [each?] paper."
I simply don't perceive that research necessarily has or should have a singular 'main message'. Research articles are complex things. Some can be hundreds of pages long. Some can be entirely descriptive e.g. taxonomic papers. The idea that research papers should be massaged to have one clear takeaway 'main message' is harmful to robust reporting of reproducible research, and it encourages questionable research practices i.e. to 'sharpen the narrative'. At the very least, I think the authors of this manuscript should just talk more about "conclusions" (plural!) and very specifically in a pluralised way. Put another way, can you recall reading a paper which performs and reports only one test? I cannot, if they exist they are super-rare. Most papers test a multiplicity of things and it is up to the reader to decide which of the tests reported are the 'main' for them and their purposes.

We agree that research papers often do not have single conclusions and have altered our wording to reflect this. The last line now reads “Moreover, the conclusions of 7.2% of non-COVID-19-related and 17.2% of COVID-19-related abstracts undergo a discrete change by the time of publication, but the majority of these changes do not qualitatively change the conclusions of the paper“.

Likewise I also wonder about the word 'reverse' in abstract. What does 'reversal' mean in this context? It appears to be assumed that all readers will have a shared understanding of exactly what this means. It seems to imply binary states to me. But I'm not sure that all conclusions from all possible research tests are necessarily so binary.

We have removed the word “reverse” in the last line of the abstract and throughout the manuscript, replacing it with “contradicts its previous version.”

For the sake of precision I must insist that the abstract should also make it clear that only bioRxiv and medRxiv preprints were sampled in this analysis. These preprint servers are not the only recognised preprint servers with COVID-19 related content out there. Moreover there are plenty of researchers e.g. in the phylodynamics community who aren't even using formal preprint servers and instead are just putting up manuscript drafts (?are these preprints?) on https://virological.org/

We have included this information in the abstract to make it clear that we only analysed data from these two preprint servers. We have added the following to the abstract “We assessed preprints (from bioRxiv and medRxiv) that had been posted and subsequently published in a journal through 30^th^ April 2020…” We would be of the opinion that data or drafts posted on sites like virological.org are preprints if they are complete manuscripts (i.e. background, data and a discussion of that data).

I also note that no mention of the choice/restriction to just bioRxiv and medRxiv prerprint servers is given in the "Parameters and limitations of this study" section. This is very much an arbitrary parameter choice and limitation to the study and so should also be discussed in that section too, even if just a few words given to acknowledge it.

We have added this information to the limitations section which now reads “Firstly, we analyzed only bioRxiv and medRxiv, and many preprints appear on other servers” (https://github.com/nicholasmfraser/covid19_preprints).

**Comments on methods**

Line 418-419 In the context for recent initiatives such as I4OA (initiative for open abstracts), it might be interesting to report the number of abstracts out of 200 which were not available via the Crossref API.

We only manually added abstracts for those articles published in more clinically orientated journals which utilized a “Dear Editor” letter format for articles. We noticed that the first paragraph immediately following “Dear editor” was the preprint abstract. However, because of the format change we decided to exclude these from our final analysis.

Statistical reporting: for tests I see that degrees of freedom and p-values are reported, but there are a few things that could have been made more explicit
e.g. for the following line:

"…a statistically significant increase compared to the 3.0% of non-COVID-19 preprints that were published (Chi-square test; χ2 = 6.77, df = 1, p=0.009) (Fig. 1A)."
I guess it is implicit that your chosen significance level (α) is 0.05? Perhaps explicitly state it?
What are the sample sizes for this test (and other tests reported in the manuscript)? I don't doubt it is reported somewhere in the manuscript but since the sample sizes vary depending on which exact test performed, the specific inclusions and exclusions, I do think it is good practice to report the sample sizes directly with the reported results so there is no chance of confusion / maximum clarity. Figure 1A doesn't help much because it also only has percentages - I presume the Chi-square test here was performed on the actual frequencies and not percentages? Perhaps put the absolute numbers on the figure, not just percentages alone?

We now specifically state the significance level within the methods and our dataset has been simplified to n = 184 for all statistical analyses, except the quoted section above which was performed on all available preprints, and we have provided a supporting n to this statement. For this Figure 1A, the Chi-square test was performed on frequencies; we have now provided those absolute numbers within Figure 1A.

Could you elaborate on the experimental design a little more from a statistical power perspective? i.e. does the seemingly arbitrary choice of sample sizes of 99 non-Covid and 101 Covid matched preprint-paper pairs, give enough statistical power to draw conclusions from? Put your calculations in the manuscript to better justify the sample sizes used. Post hoc power analysis is better than no power analysis whatsoever, although of course in an ideal world it might have been nice to preregister this research. Consider preregistering your experimental design and hypotheses to test perhaps for next time?

We now provide some basic benchmarked power calculations within the new Appendix S1 and reference this within the Methods. We calculate power for statistical tests commonly used in the manuscript applying different potential effect sizes, though we note calculation for Mann-Whitney and Kruskal-Wallis tests would require prohibitive assumptions about the underlying distributions. We also note that post-hoc power calculation using our own observed effect sizes would not be informative as simulations regularly demonstrate this to only reflect the observed p value (see <https://aurora-mareviv.github.io/talesofr/2020/01/playing-with-post-hoc-power/>). We appreciate the suggestion of preregistered research for our future investigations.

Firstly I assumed the sample sizes were 105 (covid) + 105 (non-covid), but then I saw it was 101 (covid) + 99 (non-covid) =200

But then I see this written at line ~370 "one out of 185 papers in our analysis reversing the conclusion made by its preprint", 185 is not 200. Please explain/clarify that denominator as it is a super interesting statistic but not one I trust much until I better understand its provenance.

Our original manuscript used different samples for individual analyses based on specific criteria (for example the presence of absence of abstracts, whether the manuscript had been withdrawn etc). We have now simplified this, clarified in the text and removed pairs for which we don’t have a complete analysis for all of our investigations (thereby enabling more direct comparisons between our different analyses). We now have 184 preprint-paper pairs for all analyses. We have also clarified our window for data collection.

To improve reproducibility, please state the exact versions of the software you used e.g. for rcrossref . This information was not found in either the manuscript main text, nor in the reference list where it is merely given as:
35. Chamberlain S, Zhu H, Jahn N, Boettiger C, Ram K. rcrossref: Client for Various "CrossRef" "APIs." 2020. Available: https://CRAN.R-project.org/package=rcrossref

the python SequenceMatcher is contained within the python difflib module, so you should probably cite and give the version information for difflib
likewise more precise information on the software used to implement the Microsoft Word track changes algorithm should be given (Microsoft Word? if so which version!)

We have added the Microsoft Word version number to the text and the version of Python and difflib used. We have added the crossref version used to the methods section

With regards to the R packages utilised, this data was derived from our previous work (Fraser et al, 2021) as stated in the methods section, “Our preprint dataset is derived from the same dataset presented in version 1 of Fraser *et al* [11].”

Why were these two specific algorithms used (sequencematcher and MS-Word track changes), how do they compare to the wide panoply of other algorithms used for text similarity comparison out there? This is rather undiscussed... Please cite some more papers to acknowledge the other algorithms and research on text similarity out there, and perhaps feel free to admit the choice was just convenience and familiarity (if it was. Alternatively convince us _why_ these two are the best to use for comparing abstract texts).

We have added a sentence to clarify that, for convenience, we used Microsoft Word’s merge documents feature to aggregate annotations from different annotators into a single document. We have also added a passage discussing the fact that there are many methods and implementations for measuring text similarity and we have focused on comparing only these two because we were familiar with their functionalities and type of output.

**Comments on "Parameters and limitations of this study" **

Great that you have such a section in your manuscript. Not everyone does.

I am surprised that there are no remarks given in this section as to the correspondence between mere abstracts, and the entire full text of a published research article. Much of the language and methodology in this research revolves around abstracts being representative of "main conclusions". As I have previously said research articles are long and complex things and by doing analyses of abstracts and the number of figures/tables you're really only skimming the surface of them. The analysis of abstract text-only was a design choice, and it should be discussed that this manuscript _could_ have used the full text of the research paper like the cited Nicholson et al "Linguistic Analysis of the bioRxiv Preprint Landscape" preprint. There are factors that make full-text analysis difficult e.g. that not all research papers are openly available, but interestingly that is also the case with abstracts (not all are openly available at CrossRef). I would like to see a little bit of acknowledgement of uncertainty around exactly whether abstracts always contain all the "main conclusions", are abstracts always well-written? Are all hypotheses tested in a research paper ALL reported in an abstract (no, they are not). There is certainly information loss occurring by only using abstracts is what I'm saying, and the "main" -ness of conclusions represented within the tight word-limit confines of an abstract is subjective.

We have added a sentence reading “At the same time, they are brief enough to facilitate manual analysis of a large number of papers,” to indicate that limitations of manual analysis influenced this choice. We have also added the following to the limitations section “Additionally, a study assessing whether all major changes between a preprint and the final version of the article are reflected in changes in the abstract is necessary to further confirm the usefulness of examining variations in the abstracts as a proxy for determining variations in the full text”.

Consider a discussion of the text similarity algorithms used, and those not used here too.

Towards the end of the discussion we highlight that a limitation of focusing on string similarity measures alone is that it might over-count papers that have gone through large re-arrangement without capturing whether the core of the study has remained the same or not. Recent research at the intersection of Scientometrics and Natural Language Processing is exploring whether distributional semantics methods could address this challenge. We have made this passage more explicit.

All in all this is great work and most of my comments are mostly just trying to encourage better precision and clarity.


Reviewer #2:

Learning more about preprints during the COVID-19 pandemic seems to be a worthwhile endeavor. Whether the advancements of this manuscript over prior or related research are sufficiently noteworthy for publication might lead to subjective answers - and either view would seem possible to me. In my opinion, improved statistical rigor (improved reporting on uncertainty and considering non-COVID-19 literature from same time window as COVID-19 literature) and a more conservative interpretation of their findings would seem essential to prevent misreading several of their findings and to ensure that claims are valid.

Personally, I was most intrigued by the absence of large effects, which could hint at preprints or pre-publication review doing little to better the quality of research articles. Along this line, I was left wondering whether the study suggests that also the peer review through journals has little effect on the published work aside from possibly acting as a filter for publication. Limiting the appeal of such an interpretation, the authors however discuss caveats that caution against considering their study as a measure of changes introduced by the peer review process.

We thank the reviewer for their time and comments. We purposefully decided not to attempt to draw any conclusions on the effectiveness or lack thereof in peer review as we did not directly assess this. To better answer this point, we would require access to peer-review reports, to assess what is being asked and if authors are doing all they are asked, and additional controls.

We have applied appropriate statistics throughout the manuscript and have altered the title to better reflect our narrow focus (i.e. preprints published early in the pandemic). We have additionally altered wording throughout to be mor conservative with our interpretations.

In my view the readability and usefulness of the manuscript could be extended greatly by including confidence intervals to the figures (e.g.: error bars to barplots, and notches to boxplots). The manuscript of Polka et al. inherently inspects a finite site of preprints, and - for understandable reasons - sometimes investigates only a subset of these. While I do not see this as an inherent limitation of the study given its targeted scope, small numbers will reduce statistical power. Currently, it is not clear from figures (or text) to which extent reports on presence and absence of a statistically significant different are due to effect sizes or other factors that will result in uncertainty in the analysis. Similarly, when statistical differences are claimed, it is not clear if the effect sizes could possibly be quite small. Confidence intervals for count-based data could possibly be obtained by bootstrapping, and some visualization software packages such as Python's Seaborn would already provide that option.

We appreciate the suggestion of presenting uncertainty within our figures. However, we assert that our frequency and proportion data are intended to be descriptive; reporting bootstrapped error around counts would be unconventional and we would not want to risk potential reader misinterpretation of error bars as implying these values are derived from a specific underlying model rather than descriptive summaries.

Any cases with estimated central tendencies where confidence intervals would be most appropriate were already presented as full distributions or boxplots, though we now have add notches as suggested where visually possible to do so (i.e., 95% confidence interval for medians is within IQR) and boxplot style has been made consistent. Regarding effect sizes, we report differences in absolute terms where most relevant, e.g., “with a median delay of 19 days (IQR = 15.5), compared to 101 days (IQR = 79)”, as a directly interpretable measure for the reader (noting that the actual importance of such a difference may differ drastically between fields). However, we do accept that this study has limited statistical power and we have now added calculation and discussion of statistical power in Appendix S1 (see response to Reviewer 1).

I believe that the text would need to be more careful when presenting their study as evidence against the association of preprints with "low quality", although I believe that it is fair to conclude from their data that most preprints have a similar quality to most published articles. First, the rejection of manuscripts for publication could serve as a filter against "low quality", and the present study does not investigate unpublished preprints. Second, journal reputation might signal the extent of quality - whereas this indicator is missing for preprints. Third, media coverage is not equally concerned with all preprints - thus leaving the possibility that a small number of "low quality" preprints could disproportionally affect public perception about scientific topics, including COVID-19.

We have reframed the discussion of “quality” in terms of “reliability” since, by definition, preprints can change prior to their final version. We agree that non-published preprints would be highly interesting to investigate, perhaps by comparing different preprint versions or the context of citations of those preprints in other work, but this is outside the scope of the present study.

The text should be more explicit on the time windows considered for preprint publication dates and the last date considered for journal publication. Specifically, Supplemental Figure 3F suggests that the time window could be larger than the 4 months stated in the text.

Our original manuscript used different samples for individual analyses based on specific criteria (for example the presence of absence of abstracts, whether the manuscript had been withdrawn etc). We have now simplified this, clarified in the text and removed pairs for which we don’t have a complete analysis for all of our investigations (thereby enabling more direct comparisons between our different analyses). We now have 184 preprint-paper pairs for all analyses. We have also clarified our window for data collection (Jan-Apr for COVID-19 preprints and September-Apr for non-COVID-19 preprints). The reasoning for these different time windows was due to fewer non-COVID preprints being published within this period, as mentioned in our methods section: “We generated a control set of 105 non-COVID-19 preprint-paper pairs by drawing a random subset of all bioRxiv and medRxiv preprints published in peer reviewed journals, extending the sampling period to 1st September 2019 - 30th April 2020 in order to preserve the same ratio of bioRxiv:medRxiv preprints as in the COVID-19 set.”

Further, it is my understanding of the methods section that the non-COVID-19 literature included the time frame of the COVID-19 literature, but also an equally long duration that preceded the onset of the time frame of the COVID-19 literature. Given their own data of Fraser et al., one would anticipate that the preprints in those two different observational periods behaved differently. Thus, the manuscript plausibly does not allow to conclude whether claimed differences between "COVID-19" and "non-COVID-19" are due to COVID-19 or due to broader observational window of "non-COVID-19" that additionally includes times prior COVID-19. If different time windows were used for "COVID-19" and "non-COVID-19" I would suggest redoing the analyses with a consistent time window.

Our window for data collection did differ between COVID and non-COVID (Jan-Apr for COVID-19 preprints and September-Apr for non-COVID-19 preprints). The reasoning for using these different time windows was due to the posting of fewer non-COVID preprints within this period, thereby preventing us from having a sufficiently large control dataset. Moreover, we maintained the ratio of bioRxiv v medRxiv preprints due to potential differences from the type of preprints posted to these sites. We have made this more explicit in the text.

Unfortunately, redoing the analysis with a consistent time window would suffer from the aforementioned sample size issue. The alternative would be to update the analysis for a longer time window. However, this represents a significant amount of additional work and would expand the scope of the study beyond the immediate, initial, response to the pandemic.

Despite related work from Carneiro et al. 2021, it is unclear to which extent "data availability" is a more general surrogate for "quality". Plausibly, many publications could be seen as being high quality, even if data is not available (e.g.: see Stodden et al. 2018 on study of Science, a journal enjoying a high reputation). I would advise for a more specific or weaker language.

The reference to Carneiro is in the context of “quality of reporting,” and the paragraph concerns overall changes in manuscripts. We have altered the argument for investigating the data availability to… “Data availability is a key component of the open science initiative but journal policies differ in the requirement for open data. Moreover, evidence suggests that non-scientists are utilising underlying raw data to promote misinformation”. With this framing we believe that the availability of data is now made more pertinent to the question of overall “quality” and better explains why we investigate this point.

When referring to comparisons, adding significance values to text would be necessary to see if claims are supported. One instance where it seems critical for the understanding is line 124f where the statement of specific type of change (14% vs 5%) does - in contrast to the opening statement of any change - not provide statistical support.

We have amended this section to specifically test for an association between adding any additional author and COVID-19 or non-COVID-19 preprint topic, to relate to the given percentages. We have further added supporting statistical tests throughout the manuscript.

Throughout the manuscript, it is unclear whether differences between COVID-19 and non-COVID-19 could be partially explained by research fields - or bioRxiv categories as a surrogate for the latter. Maybe it is possible to address this briefly for 1-2 claims, or by doing a supplemental analysis based on a single bioRxiv category, or by discussing it more explicitly when interpreting the data.

Although most of the 48 bioRxiv and medRxiv categories included too few preprints to analyse, we have now included a new figure and accompanying analysis quantifying our automated and manually rated changes for a subgroup of preprints within the ‘infectious diseases’ and ‘epidemiology’ medRxiv categories. We find again that COVID-19 preprints showed greater automated change measures than non-COVID-preprints, and proportions of each manual rating are consistent with our full sample (Supplemental Fig. 4)

The section starting on line 130 does not seem necessary and lacks statistical support and does not allow readers to estimate, whether effects partially stem from different volumes of COVID-19 and non-COVID-19 research. Possibly the number of articles per journal is too low beyond an anecdotal conclusion that a wide variety of journals has published ~2 manuscripts that were also preprints. In contrast, the statement on clinical or multidisciplinary journals seems intriguing, but would need to be supported by a dedicate analysis (e.g.: classifying and pooling journals, and adding statistical analysis).

We added a brief mention of these panels to the text, remaking on the breadth of journals in which the final papers appear.

The statement "Moreover, for individual preprint-published pairs, we found that there was greater variation …. (Figure 2B)" does not seem evident from the referenced panel - especially as the span is greater for non-COVID-19 in bioRxiv and the shown distribution seems to have gone some smoothing. In addition to providing statistical support, I would suggest visualizing the absolute delta of panels and tables as a boxplot that also shows the interquartile range (or even better: a letter-style plot that shows several quantiles, or as a cumulative / survival plot).

We selected violin plots because other visualisations of this distribution, including boxplots and letterplots, were not particularly informative (the vast majority of pairs had zero change in panels and tables). This has now been replaced with a dotplot for simplicity, though we maintain the difference between addition and removal of figures is an important one to distinguish in showing these data. We have now removed the statement about greater variation referencing Panel 2B, though retain the statement about greater variation within bioRxiv preprint-paper pairs with a supporting test.

Panels Figure 2D, E do not seem to be referenced or discussed in the text.

We have now added a discussion to these figures on line 194.

It is unclear whether the computational approaches to measure textual differences would be affected by text length or the adoption of "Background/Results/Discussion" subheadings that exist within the abstracts of a minority of journals.

We have added a sentence to the Limitations section, line 632: “Our automated analysis was affected by formatting-related changes in abstracts, such as the addition or removal of section headers to the abstract.”

The authors do not provide a quantification into whether the results of Figure 3A would be statistically significant.

We have now added a supporting statistical test to this statement and provide boxplot notches as suggested by the reviewer.

The caption of figure 3 reads "preprint-publication abstract pairs are not significantly different", but panel 3A and the accompanying text "we found that COVID-19 abstracts had more changes than non-COVID-19 abstracts" seem to suggest the presence of significant differences.

The caption now reads “Preprint-publication abstract pairs have substantial differences in text, but not interpretation” to reflect the detectable differences in text via automated means but absence of major changes in manual annotation.

The data shown in Supplemental Figure 3F toward delays between article publication days and preprints sometimes shows a delay in the proximity of ~10 days, which is a fast turn-around time for peer review and editorial work. This invites the question whether - as the authors briefly discuss in the discussion sections - the findings of Polka et al. truly reflect on the course between preprints and journal publication, or whether there are many manuscripts that only get posted as preprints once the journal signaled acceptance. While the authors might not be able to resolve this, I would advise to present this alternative reading more prominently and/or to try to include an analysis to address above.

We cannot definitively comment on this process as we do not have information on when individual authors are choosing to post preprints, although data in Sever et al would suggest that most authors post preprints at the time of submission (or did prior to the pandemic) with only a minority posting after receiving reviewer comments (3%). We have added this to the discussion “In a survey of bioRxiv authors, 86% reported posting before receiving reviews from their first-choice journal, but others report posting after responding to reviewers’ comments or after journal rejection [4].“ to make this point more prominent.

Figure 4 A-D are very exciting as they seem to defy the expectation that twitter and comments on preprint servers would provide feedback used by the authors. In my view this is the most novel and intriguing finding of the manuscript - even though it is discomforting as it implies that pre-publication discussions do little to improve the quality of science.

As Figure 4 A-D run against plausible anticipations or hopes toward preprints, a slightly more extended analysis would seem useful to contextualize their findings. First, what is the difference between COVID-19 and non-COVID-19 manuscripts. Second, how would preprints that are not accepted for publication compare.

We agree that it would be highly interesting to investigate those preprints that are never published. We are currently working on a separate project to investigate this question. Due to the focus of this paper, we could not investigate non-published preprints within the time period of our analysis. Moreover, to be certain that these preprints truly go unpublished, we would need a significantly longer time frame.

In response to the first point, we have investigated COVID v non-COVID as suggested (below). This highlights the discrepancy in attention that COVID preprints received over non-COVID (as we have previously published <https://journals.plos.org/plosbiology/article?id=10.1371/journal.pbio.3000959#abstract0>). We decided to combine the data for this figure in the manuscript due to the relatively low sample sizes for some categories – for example, only 1 non-COVID preprint received comments in our sample. However, we adjust for COVID-19 status of preprints in our added regression analysis to predict usage metrics (see next comment), which still supports our conclusions that the amount of attention on Twitter or number of comments is only very weakly associated with how much a preprint changes upon publication.

Previous work has indicated that, for the most part, preprint comments tend to be largely insubstantial (<https://www.biorxiv.org/content/10.1101/2020.10.14.340083v1>). Furthermore, from this analysis and our previous work, it would appear (anecdotally) that very few preprints receive “peer review” type comments. Of the discussions that are akin to peer review, these are mostly restricted to preprints receiving a high amount of attention. As such, it is not the number of comments or tweets that is pertinent but rather the content. Unfortunately, this proved beyond the scope of our work here but represents a highly interesting future avenue for investigation.

The findings of Figure 4E on the relation between citations and extent of changes make me wonder, whether they also hold when controlling for the month of the posting and/or publication.

We now provide a further statistical analysis in multivariate regression to understand whether degrees of change are associated with usage metrics when adjusting for other factors, including date of posting (measured as time since posting in days). Although we find no association is retained between citation counts and manually scored degree of abstract change in this analysis, an association is present with an automated change ratio. We discuss this in an expanded section of the results, including Table 1.

Extending beyond Figure 4E and returning to earlier panels on main findings (Figure 1D, 2C, 3C), it would be interesting to see data broken down by months (e.g.: was initial month of pandemic very different from later ones …).

Our previous work would suggest that preprints posted during the early phase of the pandemic are roughly similar in terms of attention received, citations and downloads and time to publication as well as length (Fraser et al, 2021). As we only investigate the first 4 months, we do not believe it to be beneficial to investigate our data broken down by months due to the limited sample size in each condition/month. However, we have included a preliminary analysis of the main body changes (figure content) and abstract changes here. Perhaps the most interesting insight from this analysis is the comparability between pre-pandemic and pandemic preprints.

The discussion section mentions the "beneficial effect upon preprints". While one might spot these by comparing effect- and effect+ in Figure 3F, effect sizes are small and no statistics are provided. I believe it would be safer, and more justified to the manuscript, to refer to other publications in case that the authors want to discuss positive aspects of preprints.

Due to small sample sizes, as noted, statistics would be difficult. Moreover, individual changes cannot be considered as independent, further complicating any statistics. As such we have added references to support this point (Carneiro et al and Klein et al). We have also added the following sentence to expand this point “(for example by increasing sample sizes or statistics or by making author language more conservative).”

Along this line of reasoning, I was most impressed by the apparent absence of major effects of preprints and peer-review toward the final accepted manuscript and the absence of detectable associations between comment/tweet numbers and the extent of changes. Although this might be seen as a negative finding, and may not be the findings that many scientists (and peer reviewers) would personally like to have seen, I would enjoy a further discussion of this point and would be curious about the authors' thoughts.

We have added the following to our discussion to address this “Interestingly, we did not find a strong correlation between how much a preprint changed when published and the number of comments or tweets that the preprint received (Fig. 4). This may suggest that preprint comments are mostly not a form of peer review, as supported by a study demonstrating that only a minority of preprint comments are full peer reviews [25]. Additionally, as we have previously shown, most COVID-19 preprints during this early phase of the pandemic were receiving a high amount of attention on Twitter, regardless of whether or not they were published [11].” Of note, our regression analysis does reveal a low association between change ratios and comments/citations. We suspect that a combination of all COVID preprints receiving high attention and preprint comments generally being insubstantial is the reasoning behind this lack of correlation.

According to methods section, the maximal number of figures and panels was capped at 10 and the number of supplemental materials capped at 5. While understandable, this could lead to the share of preprints without change having been overestimated (e.g.: if lengthy preprints with would undergo changes).

We hit this cap for only 6 manuscripts within the dataset, suggesting that any effects on the share of preprints with changes would be minor.

Other:

Line 95: The sentence on "At the time of our data collection in May 2020, …" is a bit unclear regarding which months were considered for COVID-19 and non-COVID-19 literature, and might even be misleading if my reading of the methods section dedicated to non-COVID-19 literature was correct.

All data was collected on 1^st^ May 2020. However, the time windows for COVID v non-COVID did differ and we have now explained this in text and previous responses here. We hope this clarifies our manuscript. Our methods section also clarifies the data collection windows “We generated a control set of 105 non-COVID-19 preprint-paper pairs by drawing a random subset of all bioRxiv and medRxiv preprints published in peer reviewed journals, extending the sampling period to 1st September 2019 - 30th April 2020 in order to preserve the same ratio of bioRxiv:medRxiv preprints as in the COVID-19 set”

Panel 1B could go further. The main impression from the panel is that few journals do open peer review. This might not be surprising. However, the figure (and text) does not proceed to investigate two points that would seem to add to the main narrative. First, are there statistical differences between COVID-19 and non-COVID-19 (e.g.: the results could be alternatively visualized to show that in non-COVID-19 transparent peer review is ~5 times higher than in COVID-19, which might have interesting/discomforting implications on openness or rigor of COVID-19 literature). Second, although only a subset of articles has transparent peer review, the authors do not report on whether that reduced number would still have sufficed to address their initial goal of estimating quality. Additionally, several publishers offer peer reviewing data to scientists performing meta-research - which might have enabled the authors to proceed toward their investigation of "quality" even if the public data was scarce.

We have now expanded the text to compare the percentages of COVID-19 and non-COVID-19 preprints with transparent peer review, and a supporting statistical test (in which we do not find strong evidence of association). Unfortunately, we did not seek peer review data from publishers due to difficulties in obtaining related data for use in previous project. Furthermore, we have altered our wording of “quality” to “reliability” throughout the manuscript to better reflect that we do not assess the quality of the science, rather the comparability of preprints to their published counterparts.

The statement on line 160 might not be "surprising" given Adams et al. 2021, Nature.

We have removed the word “surprising” from this statement.

Neither text nor supplemental method 3 seem to indicate what "Microsoft word track changes algorithm" measures. Especially, it is unclear to me how to interpret scores above 1.0 (e.g.: would 1.1 and 0.9 be equally close to large rewrites as equidistant from a value of 1.0, as suggested on line 188).

We have expanded our explanation of this method in the methods section. Specifically, “To compute the change ratio of a pair of abstracts, following the Python implementation, the formula is 2*M/ T where M is the number of characters in common and T the total number of characters in both sequences. So the ratio will span between 1, if the abstracts are identical, and 0 if there is no snippet in common. As Microsoft Word track changes only provides statistics on the characters changed (inserted, removed, etc) but no information is available on the characters that are in common between two abstracts, we derive M by computing the total number of characters in the final abstract minus the characters that have been inserted”

Line 177: "drastically re-written" could possibly be toned down, since the reference frame for what constituted drastic re-writes might be subjective and difficult to define.

We have altered this to “substantially re-written” and qualify the statement with a defined cutoff of change ratio of 0.75 or greater.

Panel 3D is very difficult to read as most information is close to 0 while the text intends to convey that there are some manuscripts that mainly undergo positive or negative changes. I am unsure whether the latter warrants a main figure. Would suggest testing (and maybe include if clearer) an alternate visualization, where x-axis is given by total number of positive and negative scores, and y-axis shows in a boxplot for each preprint-publication pair, which share of the score is falls onto positive changes. Thus, one could see if there is generally more variability, or if there also is a systematic trend.

We attempted the boxplots as suggested, though we do not have enough data to meaningfully visualise boxes for each value of total score, and an equivalent scatter plot did not show these data more clearly than in Figure 3D (see below). Instead, we have now plotted these as a heatmap with numbers of COVID-19 and non-COVID-19 preprints labelled for each value of sum of positive score and sum of negative scores.

While the paragraph dedicated to Supplemental Fig 3G is somewhat interesting, the absolute number of articles is small (generally ranging between 1 and 5). Consequently, it is difficult to conclude whether the statements from the text refer to anything that is specific to preprints, or if they merely reflect on different journals publishing a different number of articles (e.g.: finding multiple articles from PLOS ONE might indicate that PLOS ONE publishes more articles than other journals).

We agree that due to the small number of preprints published by individual journals it is difficult to draw robust conclusions, and this may be reflective of different journals publishing different numbers of articles. We did touch upon this in the text “although this journal published the most observed non-COVID-19 preprints”. The primary message of Supp fig 3G is to demonstrate that (early in the pandemic) a wide array of journals were publishing preprints with no, obviously noticeable, differences in the comparability of the final published version and preprint. It is possible that an analysis at a later time period may reveal more subtle differences but this would be beyond the scope of our work here.

While the authors' attempts to use controlled criteria for the manual scoring of changes are laudable, it is unclear, whether additional measures had been taken to avoid human biases (e.g.: blinding annotators to prior results, or possible scope of publication) - especially as the discussion section notes that "different assessors may annotate changes differently". While I do not intend to suggest new analysis such a limitation could be noted in the main text.

Since each annotator worked independently, annotators were blinded to one another's interpretations during the initial analysis. However, we did discuss the given scores over iterative rounds of review to generate a consensus on each article. We have noted this limitation on lines 623-626.

To better connect Figures 3 and 4, it would be nice if an analysis along Figure 4 would also be repeated in supplemental figures for the metric of change presented in Figure 3A. Given the ordinal nature of the metric of Figure 3A, the authors could also do a scatter plot or provide a Spearman correlation (e.g.: do articles with larger textual change also get a higher number of tweets….). Adding such an analysis might also allow for a stronger discussion of differences between computational and expert-supervised approaches to identify changes.

We now compare our automated and manual approaches to measuring change by including each as predictors in our multivariate regressions predicting number of tweets, number of comments, and number of citations. We find that articles with larger textual changes do not get a higher number of tweets, but do receive more comments and citations, detailed in new results text accompanying Table 1. (Note that no multicollinearity was detected between our different measurements of change in these regressions).

The lengthy discussion of data availability (and using it as a readout of "quality") seems peripheral to the manuscript and its findings.

We have altered the reasoning for investigating the data availability to… “Data availability is a key component of the open science initiative but journal policies differ in the requirement for open data. Moreover, evidence suggests that non-scientists are utilising underlying raw data to promote misinformation”. With this framing we believe that the availability of data is now more pertinent to the question of overall “quality” and better explains why we investigate this point.

The statement on "this potential would be amplified if increasing calls for abstracts and article metadata to be made fully open access" does not flow from the manuscript and falls short to recognize that several publishers will provide researchers of science with additional data around their publications and peer review process. Hence open access is not strictly needed (although it would be convenient).

We have added an explanation that we needed to manually collect abstracts for several papers in our study, which would limit the scope of a larger-scale NLP project.

The discussion refers to "our approach revealed that computational approaches comparing textual changes at string-level are insufficient", whereas the argument to be drawn from the discussion is that computational and non-computational approaches can differ (hence either could be insufficient or complementary). Moreover, Figure 3 A-C show that overall, they don't differ in the resulting main conclusions.

We have amended this aspect of our discussion which now reads “Our approach revealed that computational approaches comparing textual changes at string-level do not predict the extent of change interpreted by human readers.”.

The authors' scoring criteria for change considers research claims, but - in possible opposition to the computational approaches - not the clarity of the text or its adjustment to a specialized group of readers. Improved communication could be seen as one of several qualities of manuscripts.

We agree that improved communication may be one area of improvement and would be an aspect of quality and this would be an interesting analysis for future work; anecdotally, this was not something that we particularly observed during these analyses (although we did not directly assess this aspect) with most changes being sample size increases or differences in tone.

Computational approaches to identify similarity between abstracts could be extended toward the recognition and differences in key concepts. While I'm not suggesting new work, there might be a way to add a half-sentence to the discussion - possibly referring to Nicholson et al. 2021 .

We have added a sentence in the discussion on this and pointing to the recent work of Nicholson et al.

The methods section lists the transformation of "fever" to "high fever" as as "substitution of a noun". While I see the underlying rationale for distinguishing "fever" and "high fever", I am unsure whether - grammatically - above is an example of the substitution of a noun. Instead, it appears to me that above is the addition of an adjective as an attribute to a noun, and might even fall into the effect+ category as "high fever" (which would still be a fever) would remove fever with lower temperatures.

We use “substitution of a noun” to indicate that some concept or measure has been replaced by another concept or measure.

Quantifying whether titles changed could allow for additional comparisons to existing research into changes of preprints (Lin et al., 2020, Scientometrics).

Titles were matched based on data provided by bioRxiv and medRxiv which means that any changes in titles would not have been included in this dataset. Due to the, relatively, low number of preprints published in this time, we do not anticipate that many pairs would have been missed by relaying on this method. However, we agree that this would have been an interesting additional analysis.

While the authors follow the reasonable reductionistic assumption that peer-review and preprints have the potential to improve manuscripts, Crane, 1967, The American Sociologist, discusses the more nuanced possibility that peer review serves to make research more conservative. Maybe the discussion section could propose ways how their strategy could be applied to investigate above.

This is an interesting idea, but as presently formulated, our strategy cannot isolate the effect of journal peer review from other factors that may influence a manuscript, such as additional feedback outside of journals or simply additional work by authors. As such, our work is focused more on comparability of preprinted work to the published version. Anecdotally, we did notice many of the changes to be altered wording that limited the authors interpretations.


Reviewer #3:

This study asks an important and salient question in scientific publishing: (how) do preprints change between their initial release on a preprint server and their eventual publication in a peer-reviewed journal? This has major implications for how we assess the quality of preprinted research, the degree to which we should depend on pre-publication peer review, and the value we assign to peer review as an academic practice, along with peer-reviewed journals as a whole. The paper is well-written and the authors have clearly gone to great lengths to curate and analyze this dataset in a way that enables an extremely thorough assessment of the changes preprints undergo on their journey to peer-reviewed publications.

My only major criticism of this study is that the non-COVID-19 articles included in the analyses are not necessarily representative of the "baseline" for how articles change between preprint and peer-reviewed publication, despite the authors seeming to make this generalization throughout the paper. Many of the non-COVID-19 articles posted during this time were likely impacted by the pandemic through lockdowns resulting in lost time in the lab, shortages and diversions of resources (including human, monetary, and lab supplies/equipment), and the broad psychological effects of the pandemic. In addition to many journals expediting peer review of COVID-19 articles, they also often generous extensions to reviewers and authors of non-COVID-19 submissions and reviewers were encouraged to be more lenient in requesting new experiments, etc. that could not feasibly be completed due to lockdowns. These factors undoubtedly could have an impact on many of the metrics evaluated here (e.g., time to publication, evidence of additional experiments, etc.).

Ideally, this study would be revised throughout to consider a third group of preprints posted before the pandemic for comparison, e.g., those posted between Jan-Apr 2019, but I recognize that this is likely a massive amount of work and would only consider it worthwhile if the authors feel confident it could be completed within a typical response window. Instead, I would suggest the authors carefully go through the manuscript and ensure that they are not overstating the scope of their study and conclusions (the title may also need to be revised to reflect this limited scope). Alternatively, I wonder if the non-COVID-19 preprints could be analyzed in separate groups, one from 1 Jan - 11 Mar, 2020 (before the WHO announced the pandemic) and the other from 11 Mar - 30 Apr 2020.

We would like to thank the reviewer for their comments and time.

We appreciate the suggestion of a third group of preprints posted prior to the pandemic, however, as the reviewer notes this would be a large amount of extra work. We are currently planning a more thorough investigation of preprints not specifically coupled to any pandemic, including preprints that are never published, but this is a major undertaking and the subject of a separate study. This current work was focused on the how preprints changed during the early phase of the pandemic, when many were raising concerns over the usage of preprints. We have altered our text to make the lack of a true baseline clearer and added the following to our limitations section to better reflect that publishers had adopted altered practices during this period “Finally, our non-COVID-19 sample may not be representative of “normal” preprints, as many aspects of the manuscript preparation and publication process were uniquely affected by the pandemic during this time.” We have also altered the title to better reflect the focus of this work. The title now reads “Preprints in motion: tracking changes between posting and journal publication during a pandemic”.

With regards to separating our data into two groups, we do not have a large enough sample size to draw adequate conclusions if we were to do this. However, by restricting our interpretations and being clearer on our focus we believe we have addressed this issue.

Minor comments:
Line 102-103: I wouldn't necessarily claim that medical/epidemiological medRxiv preprints are "more relevant to the pandemic" than, say, bioRxiv preprints in evolutionary biology attempting to determine the origin of SARS-COV-2.

We have removed this statement from the text.

Line 112-114: While I fully agree that requiring underlying data to be accessible is an important mechanism to promote transparency and establish confidence in published research, this section seems to conflate the actual quality of the research with the ability to assess that quality because data are available. A reduction in data availability between preprint and peer-reviewed forms of a paper is not evidence of a reduction in quality (and vice versa). Further, given that data availability (or lack thereof) seems to be mostly a matter of different standards/expectations between fields/journals, I'm not convinced changes to data availability are a meaningful measure of differences between COVID-19 and non-COVID-19 preprint-publication pairs.

We have altered the reasoning for investigating the data availability to… “Data availability is a key component of the open science initiative but journal policies differ in the requirement for open data. Moreover, evidence suggests that non-scientists are utilising underlying raw data to promote misinformation”. With this framing we believe that the availability of data is now more pertinent to the question of overall “quality” and better explains why we investigate this point.

Line 122: This could use a citation.

We have altered our wording so that this is a less definitive statement: “The number of authors may give an indication of the amount of work involved; we therefore assessed authorship changes between the preprint and published articles.”

Line 127: I'd also be interested to see, among the ~25% of papers with authors added, how many authors were added, and if this also correlates with time to publication and/or significant changes to the text of the paper.

There were only 21 preprint-paper pairs for which authors were added which limits how useful any further interpretation of this data would be. We have gone back through this data and quantified precisely how many authors were added in each pair. For the vast majority (~60%) only 1 author was added to the paper, with ~15% having 2 authors added. However, these numbers are too small to infer meaning from.

Line 177: Can you include the exact value of "sizable number" in the text?

We now refer to the specific number of preprints having a given level of change ratio.

Line 220-222: Based on conversations with colleagues involved in COVID-19 research, my impression is that the tendency of these papers to update with larger sample sizes ultimately boils down to reviewers/editors requesting the analyses be repeated with new additional data (especially among epidemiological studies), due to the pandemic's rapid progression. Could this be broken down by subject area? If there are differences, this could be useful to COVID-19 researchers in isolating which subject areas are likely to request additional data in peer review and preemptively preparing for such updates.

As detailed in our response to Reviewer 2, we have now included a subgroup analysis for several pertinent categories of research relevant to COVID-19: ‘infectious diseases’ and ‘epidemiology’. However, we do not find an increased tendency in this subgroup for changes to include new assertions or changes in statistical significance compared to our whole sample (Supplemental Fig. 4).

Line 237-239: it seems highly counterintuitive that non-COVID-19 articles with major changes did not experience significantly longer delays than those with no change. Is this simply due to lack of power because there were so few articles with major changes?

We have clarified in text that our limited sample size for major changes limits the interpretation of this piece of data “though we only observed seven such articles which limits the interpretation of this finding”.

Line 274-275: it should be trivially easy to test with regression or ANOVA whether the increased citations of preprints undergoing discrete changes is in fact associated with a lengthier peer review time (or, more precisely, time between preprint and peer-reviewed publication).

We include delay between preprint posting and publication as a covariate in a new regression analysis predicting citations among other usage metrics, as suggested (Table 1). We find that some measures of discrete change to abstracts persist even when adjusting for this delay, and that generally, COVID-19 status and overall time since posting were much stronger explanatory factors in preprint usage.
